# Effects of different mulching practices on soil microbial community structure, function, and interaction networks in a chieh-qua cultivation

**DOI:** 10.3389/fmicb.2026.1691984

**Published:** 2026-02-04

**Authors:** Yan-chun Qiao, Xiao-xin Jiang, Jian-po Zhan, Xiao-hui Cheng, Feng Liu, Wen-sheng Zhang, Guo-ping He, Jia-zhu Peng, Yu-jun Wu, Song-guang Yang

**Affiliations:** 1 Guangzhou Academy of Agricultural and Rural Sciences, Guangzhou, China; 2 College of Horticulture, South China Agricultural University, Guangzhou, China; 3 Guangdong Key Laboratory for New Technology Research of Vegetables, Vegetable Research Institute, Guangdong Academy of Agricultural Sciences, Guangzhou, China

**Keywords:** chieh-qua, metagenomic, microbial community, mulching, soil

## Abstract

**Background and aims:**

Mulching is a widely used agricultural management practice with profound effects on soil properties and crop productivity. However, its impact on soil microbial community structure and function remains insufficiently understood. This study aimed to investigate how different mulching treatments influence the composition, functional potential, and interaction networks of soil microbial communities in a chieh-qua–legume rotation system.

**Methods:**

Metagenomic sequencing was employed to analyze soil samples subjected to four mulching treatments (biodegradable mulch, non-degradable silver mulch, non-degradable black mulch, and straw mulch) as well as a no-mulch control (CK).

**Results:**

Mulching treatments significantly altered soil microbial diversity and community structure, with straw and biodegradable mulches supporting higher diversity than the control. Biodegradable mulch was strongly correlated with changes in soil pH and enriched denitrifying bacteria such as Thauera and Comamonadaceae, while reducing the abundance of genes related to energy metabolism and carbon fixation. These findings suggest that organic carbon from mulch degradation may enhance denitrification, potentially leading to nitrogen loss. Co-occurrence network analysis revealed that biodegradable mulch promoted more complex and connected microbial networks, whereas plastic mulches resulted in simpler structures. Additionally, all mulching treatments significantly reduced the abundance of the autotrophic ammonia-oxidizing archaeon Thaumarchaeota, likely due to reduced soil oxygen under mulch.

**Conclusion:**

This study provides new insights into how different mulching practices modulate soil microbial communities and their ecological functions. The results underscore the importance of tailoring mulching strategies to maintain soil health and fertility. Specifically, nitrogen supplementation is recommended when using biodegradable mulch in chieh-qua cultivation systems.

## 1 Introduction

Chieh-qua (*Benincasa hispida*), an important variety of the Cucurbitaceae family, is a characteristic vegetable crop widely cultivated in southern China and Southeast Asia, possessing significant economic and medicinal value (Mao et al., 2017). However, with the continuous expansion of its cultivation area and the intensification of monoculture practices, soil degradation has become increasingly severe, leading to frequent occurrences of pests and diseases. This has emerged as a critical factor limiting the improvement of yield and quality in chieh-qua production in China (Che et al., 2023). To alleviate these problems, farmers commonly adopt a chieh-qua–legume rotation system, which helps restore soil fertility and reduce disease pressure. Within this rotational system, mulching is widely used as an auxiliary management practice.

Mulching is an effective agricultural practice for improving soil quality and increasing crop yield. By covering the soil surface with plastic films or other materials, mulching helps retain soil moisture, regulate temperature, and suppress weed growth, thereby creating favorable conditions for crop production, particularly in arid and semi-arid regions (Zhang et al., 2018; Liu et al., 2019). However, conventional plastic mulches are difficult to remove and recycle after use, leading to the accumulation of plastic residues and microplastics in soils (Kasirajan and Ngouajio, 2012). This accumulation can negatively affect soil physical properties and hinder plant root development, ultimately threatening soil health and productivity (Jiang et al., 2017; Qi et al., 2020). To overcome these issues, biodegradable mulches and organic materials such as straw and plant residues have been developed as sustainable alternatives. These materials can be decomposed by soil microorganisms and have been shown to improve soil fertility and structure (Zhang et al., 2020; Qi et al., 2022).

Despite altering the physical and chemical properties of soil, mulching also exerts significant influences on soil microbial communities, which play key roles in nutrient cycling and ecosystem functioning. Previous studies have shown that mulching can modify soil temperature, moisture, and organic carbon availability, thereby reshaping microbial composition and activity. For example, plastic mulching often increases soil bacterial abundance but may reduce microbial diversity due to limited aeration and changes in carbon substrates (Kong et al., 2024). In contrast, biodegradable and organic mulches tend to promote microbial biomass and enzymatic activity by providing additional carbon sources during decomposition (Liu et al., 2023). Moreover, mulching can influence specific microbial functional groups, such as nitrifying and denitrifying bacteria, thereby affecting nitrogen transformation and greenhouse gas emissions (Xiong et al., 2023). However, the direction and magnitude of these effects vary depending on mulch type, soil conditions, and climatic factors. Despite increasing attention, the mechanisms by which different mulching materials shape soil microbial communities and their functional potentials remain poorly understood, highlighting the need for further investigation.

Mulching is an agricultural technique that involves covering the soil surface with plastic films or other materials to protect crops. This method reduces water evaporation, maintains soil temperature, and suppresses weed growth, thereby creating a more favorable environment for crop growth, particularly in arid and semi-arid regions. Additionally, mulching can reduce irrigation requirements and minimize soil water loss, significantly enhancing water use efficiency (Zhang et al., 2018; Liu et al., 2019). However, traditional plastic mulches are difficult to remove and recycle after use, leading to the accumulation of microplastics in the soil, which has become one of the primary sources of soil microplastic pollution (Kasirajan and Ngouajio, 2012). Long-term plastic accumulation can impede water infiltration, inhibit soil gas exchange, and restrict root growth, ultimately reducing soil productivity (Jiang et al., 2017; Qi et al., 2020). As a result, plastic pollution has emerged as a potential threat to soil ecosystems. Despite this, the use of agricultural plastic mulches has surged in recent years due to their significant improvements in water and nutrient use efficiency, although the recycling rate of used mulches remains below two-thirds (Kim et al., 2021; Xue et al., 2021). To address these issues, biodegradable mulches and organic mulching materials (such as straw or plant residues) have gradually become ideal alternatives to plastic mulches. Biodegradable plastic films can be incorporated into the soil through tillage, where they are decomposed by naturally occurring microorganisms and ultimately converted into carbon dioxide and water under aerobic conditions (Narayan, 2010). In contrast, the use of organic mulches stimulates the activity of soil microbial communities, enhances the organic matter and nutrient content of the soil, and additionally helps to reduce extreme soil temperature fluctuations, particularly alleviating the impact of heatwaves in high-temperature regions (Pou et al., 2021; Scavo et al., 2022).

Soil microorganisms are a vital component of soil ecosystems, playing key roles in organic matter decomposition, humus formation, and nutrient transformation and cycling (Baldock and Skjemstad, 2000). They are often used as important indicators of soil health. Different mulching practices can significantly alter the composition of soil microbial communities. For instance, Dong et al. (2017) found that plastic mulching significantly increased soil microbial diversity and richness, with profound effects on microbial community structure. Zhang et al. (2022) demonstrated that biodegradable mulching enhanced soil total nitrogen, available phosphorus, and available potassium content during garlic cultivation, while also improving soil microbial activity, urease activity, and catalase activity, thereby boosting soil fertility. Organic mulching, as another effective method for soil improvement and environmental protection, not only increases water infiltration, prevents soil nutrient loss, and suppresses weed germination but also controls crop pests and diseases while enhancing the biodiversity of soil microecosystems (Renkema et al., 2016; Jiménez et al., 2017; Yaşar Korkanç and Şahin, 2021). Studies have shown that organic mulching significantly alters the richness and diversity of microbial communities in vegetable and tea soils (Brennan and Acosta-Martinez, 2017; Zhang et al., 2020). In summary, mulching practices can profoundly influence soil microbial diversity and richness, thereby affecting crop yield. However, research on the effects of different mulching materials on soil microorganisms under chieh-qua rotation systems remains limited and warrants further investigation.

This study employed metagenomic sequencing to systematically analyze the effects of four mulching treatments (biodegradable mulch, non-degradable silver mulch, non-degradable black mulch, straw mulch) and a no-mulch control (CK) on the structure, function, and interaction networks of soil microbial communities in chieh-qua cultivation. The research revealed the mechanisms by which different mulching practices influence soil microbial diversity, functional gene expression, and key microbial taxa, and explored the potential impacts of mulching on soil nitrogen cycling and carbon metabolism. These results provide a theoretical basis for the use of mulching films in chieh-qua cultivation. By elucidating the effects of mulching practices on soil microbial communities and their functions, this study offers new insights for sustainable agricultural development.

## 2 Materials and methods

### 2.1 Trial design

All materials were provided by the Guangzhou Academy of Agricultural and Rural Sciences and cultivated at the Nansha site (Guangzhou, 23.4 N, 113.4 E, China). The soil was clay loam with the following initial physical and chemical properties: Cadmium: 0.463 mg/kg; Total Mercury: 0.15 mg/kg; Total Arsenic: 14 mg/kg; Lead: 40.5 mg/kg; Total Chromium: 74.4 mg/kg; PH: 7.5; Organic matter: 28.96 g/kg; Copper: 46.3 mg/kg; Fast-Acting Potassium: 81.1 mg/kg; Fast-acting phosphorus 23.7 mg/kg. The experiment adopted a loofah-cowpea rotation system, with *Benincasa hispida* planted in the first half of the year and cowpea (*Vigna unguiculata*) planted in the second half of the year. There were five treatments (CK, A1, A2, A3, A4) in the experiment: A1 was covered with biodegradable mulch, A2 was covered with non-degradable silver film, A3 was covered with non-degradable black mulch, A4 was covered with straw, and CK was the control without any covering material. Each treatment was repeated 3 times, and the experiment lasted for 12 months (from March 2022 to March 2023). Soil samples were returned to the laboratory immediately after collection. All samples were sieved through a 2 mm mesh, homogenized into uniform small particles, and then divided into two parts: one part was air-dried for soil physicochemical analysis, and the remaining samples were stored at −80 °C until metagenomic sequencing.

### 2.2 Soil physical and chemical properties determination

A soil suspension (1:2.5 w/v) was prepared, and the soil pH was measured using a pH meter (PHS-3C, Shanghai Yidian Scientific Instruments Co., Ltd., China). Total nitrogen (TN) in the extract was determined using an elemental analyzer (vario MAX cube, Elementar, Germany) (Watanabe and Olsen, 1965). Available phosphorus (AP) was measured following the molybdenum blue method using hydrochloric acid and ammonium fluoride (Gong et al., 2016). Additionally, available potassium (AK) was extracted with ammonium acetate and determined by flame photometry (Dotaniya et al., 2023). Soil organic matter was physically fractionated into particulate organic matter (POM) and mineral-associated organic matter (MAOM) according to the size-based method proposed by Cambardella and Elliott (1992). Briefly, 15 mL of sodium hexametaphosphate solution (5 g L^−1^) was added to 5 g of soil (< 2 mm) and dispersed in a horizontal shaker for 16 h at 140 rpm. The dispersed solution was then passed through a 53 μm sieve by gently adding distilled water. The coarse fraction retained on the sieve (>53 μm - POM) and the material washed through (< 53 μm - MAOM) were collected, oven-dried (50 °C), and ground (< 0.15 mm) for carbon determination. This process was repeated in triplicate for each soil layer as a strategy to reduce the number of samples for analysis. The concentrations of heavy metals (Cu, Hg, As, Pb, Cd, Cr) in the soil were determined using closed-acid digestion followed by inductively coupled plasma mass spectrometry (ICP-MS). The ICP-MS analysis conditions were as follows: RF power 1,400 W, sampling depth 15 mm, auxiliary gas (argon) flow rate 0.8 L/min, cooling gas flow rate 13.0 L/min, peristaltic pump speed 30.0 rpm, channel 3, with three replicates and 100 scans per sample. The instrument was optimized before sample analysis to ensure operation under optimal conditions.

### 2.3 DNA extraction, Illumina sequencing

Genomic DNA was extracted from rhizosphere soil samples using the PowerSoil DNA Isolation Kit (MoBio, Carlsbad, USA) according to the manufacturer’s instructions. The concentration and purity of the soil DNA were measured using a NanoDrop 2000 spectrophotometer (Thermo Scientific, Waltham, USA), and the DNA quality was verified by 1% agarose gel electrophoresis. PCR products were purified using the AxyPrep DNA Gel Extraction Kit (Axygen Biosciences, USA). Subsequently, PCR amplification was performed in a 20 μL reaction system using TransStart Fastpfu DNA Polymerase (AP221-02, Transgen Biotech, Beijing, China). The amplification protocol was as follows: initial denaturation at 95 °C for 3 min, followed by 27 cycles of 95 °C for 30 s, annealing at 55 °C for 30 s, and extension at 72 °C for 30 s, with a final extension at 72 °C for 10 min. The amplified DNA was quantified using the QuantiFluor™-ST system (Promega, Madison, USA). A paired-end library was prepared using the TruSeq™ DNA Sample Preparation Kit (Illumina, San Diego, USA). Adapters containing hybridization sites for the full set of sequencing primers were ligated to the blunt-end fragments. Sequencing was performed on the Illumina MiSeq PE 300 high-throughput sequencing platform.

### 2.4 Analysis of species composition in soil

To obtain high-quality clean reads, raw data from the Illumina platform were filtered using FASTP (v0.23.4) (Chen et al., 2018). The trimmed reads were then processed using Kraken2 (v2.1.3) (Wood et al., 2019) to identify the microbial composition of the community. Taxonomic abundances were re-estimated at the species level using Bracken (v2.9) (Lu et al., 2017) to generate an operational taxonomic units (OTUs) table. Rarefaction curves were plotted using the rarecurve function from the vegan package, and alpha diversity was calculated using the Shannon index and Chao1 index (Wang et al., 2007). To compare community structures across different environments, Principal Coordinate Analysis (PCoA) based on Bray-Curtis distance and Analysis of Similarities (ANOSIM) were performed. Differences at the phylum and species levels were assessed using ANOVA in the STAMP software (v2.1.3) (Parks et al., 2014), with *p*-values adjusted for false discovery rate (FDR) using the Benjamini-Hochberg (BH) method. Significant differences were identified based on an FDR threshold of ≤0.05, and the results were visualized using STAMP. Additionally, LEfSe (Linear Discriminant Analysis Effect Size) analysis was conducted on the Galaxy platform^1^ to identify features with significant discriminatory power, and the results were visualized. To explore ecological interactions among microbial taxa, SparCC3 (Friedman and Alm, 2012) was employed to construct microbial interaction networks. In brief, we first calculated the SparCC correlation coefficients for the OTU table of each treatment group and performed 100 bootstrap resamplings to assess the significance of the correlations, resulting in a pseudo-*p*-value matrix. After combining the correlation coefficients and the *p*-value matrix, correlations with SparCC ≥ 0.9 and *p*-value < 0.05 were considered significant and retained as edges connecting microbial taxa. These significant correlations (edges) and the corresponding OTUs (nodes) were then used to construct the microbial co-occurrence network. The topological properties of the networks were analyzed using the R package igraph,^2^ and the networks were visualized using Cytoscape (v3.10) (Shannon et al., 2003).

### 2.5 Functional prediction and environmental factor correlation analysis

Functional profiling of the metagenomic data was performed using HUMAnN2 (v0.11.2) (Franzosa et al., 2018). Briefly, HUMAnN2 employs DIAMOND (v0.9) (Buchfink et al., 2015) to align quality-controlled reads against the UniRef90 protein database, quantifying the abundance of each protein. Based on the mapping between UniRef90 IDs and functional database IDs, abundance tables for databases such as KEGG were generated. For KEGG Orthology groups (KOs), Principal Coordinate Analysis (PCoA) was conducted using the Bray-Curtis distance matrix combined with Analysis of Similarities (ANOSIM). To identify differentially enriched KO pathways and modules, the ReporterScore R package (Peng et al., 2024) was utilized for enrichment analysis at both the module and pathway levels. Significant pathways and modules were visualized based on their reporter scores. Additionally, we performed a Mantel test using the linkET R package (Borcard et al., 2011) to assess the correlation between soil physicochemical properties, microbial abundance, and KEGG gene abundance, in order to identify the environmental factors influencing microbial communities. Subsequently, to analyze the interactions between different mulching treatments, microbial communities, and environmental factors, we first determined the optimal environmental combination. In brief, we calculated the variance inflation factor (VIF) for environmental factors using the vif.cca function from the vegan package and conducted forward selection analysis. We then used the bioenv function to select the combination of environmental factors, combining the results from forward selection to ultimately determine the optimal set of environmental factors. Finally, based on the screened environmental factors, Canonical Correspondence Analysis (CCA) was conducted using the vegan R package to evaluate the contribution of each physicochemical parameter to variations in microbial community composition.

## 3 Results

### 3.1 Characterization of metagenomic sequencing datasets

**Figure 1:**
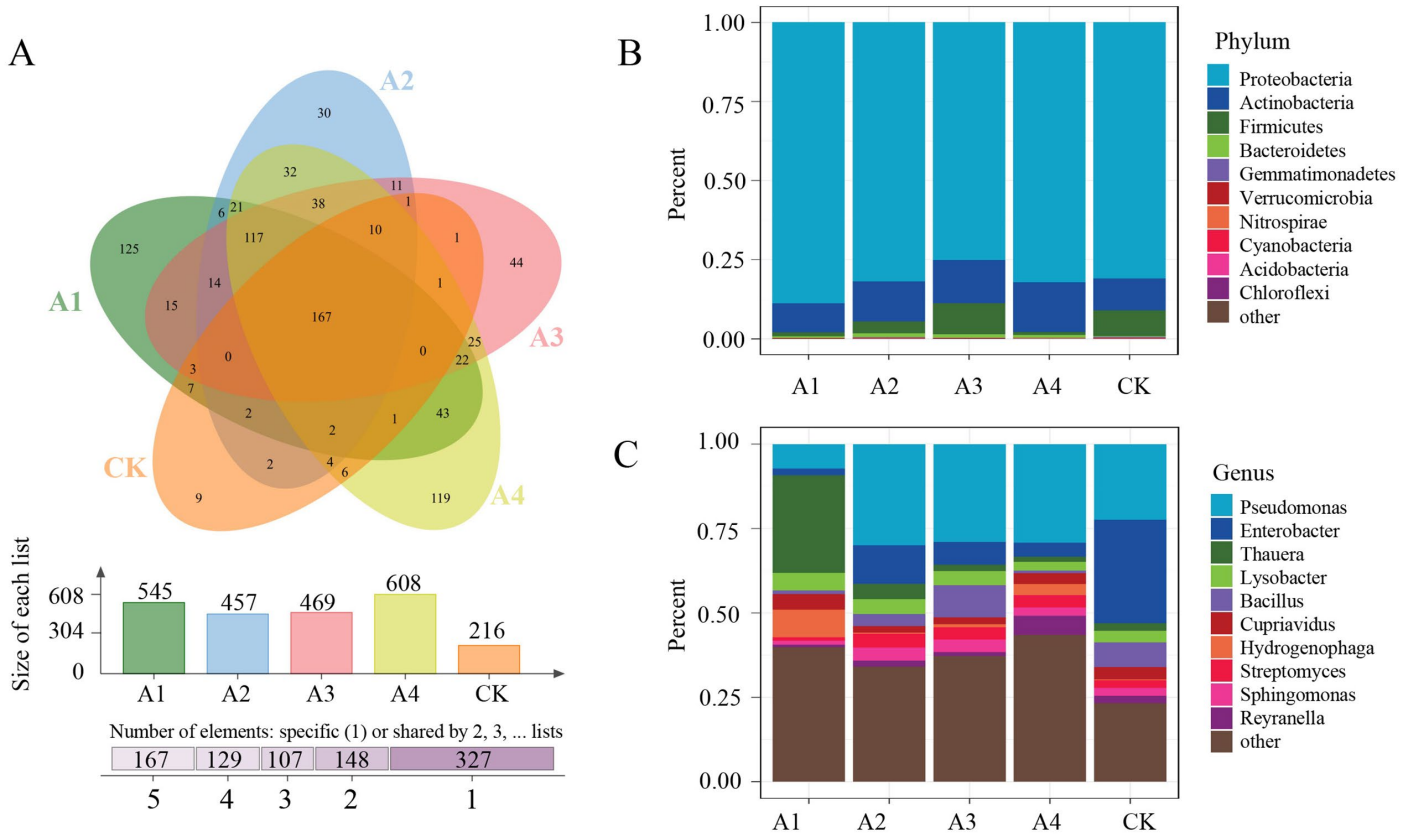
Overview of soil microbial metagenomes under different mulching practices. **(A)** Venn diagram illustrating the microbial communities in five soil samples; **(B)** relative abundance of the top 10 microbial phyla in five soil samples; **(C)** relative abundance of the top 10 microbial genera in five soil samples.

To investigate the microbial communities in soils under different mulching treatments, this study employed the Illumina platform to perform metagenomic sequencing on soil samples subjected to five distinct mulching practices (Supplementary Table S1). Based on k-mer-based Kraken2 taxonomic annotation analysis, all reads were classified into 878 OTUs, among which 167 OTUs were shared across all samples. The A1 treatment group exhibited the highest number of unique OTUs (125), while the CK had the fewest unique OTUs (9) (Figure 1A; Supplementary Table S2). At the domain level, all reads were categorized into three domains and viruses, with bacteria accounting for over 99%, and archaea, eukaryotes, and viruses collectively representing 0.67% (Supplementary Figure S1). At the bacterial kingdom level, 851 bacterial species spanning 16 phyla were identified, with Proteobacteria (81%), Actinobacteria (13%), Firmicutes (4%), and Bacteroidetes (1%) showing the highest relative abundances. Notably, the A1 treatment group exhibited the most prominent relative abundance of Proteobacteria (Figure 1B). At the genus level, 10 genera, including *Pseudomonas*, *Enterobacter*, *Thauera*, *Lysobacter*, *Bacillus*, *Cupriavidus*, *Hydrogenophaga*, *Streptomyces*, *Sphingomonas*, and *Reyranella*, displayed relatively high abundances. Among these, *Thauera* was most abundant in the A1 treatment group, *Pseudomonas* dominated in the A4 treatment group, and *Enterobacter* showed a higher relative abundance in the control group (Figure 1C).

### 3.2 Soil microbial diversity under different treatments

**Figure 2:**
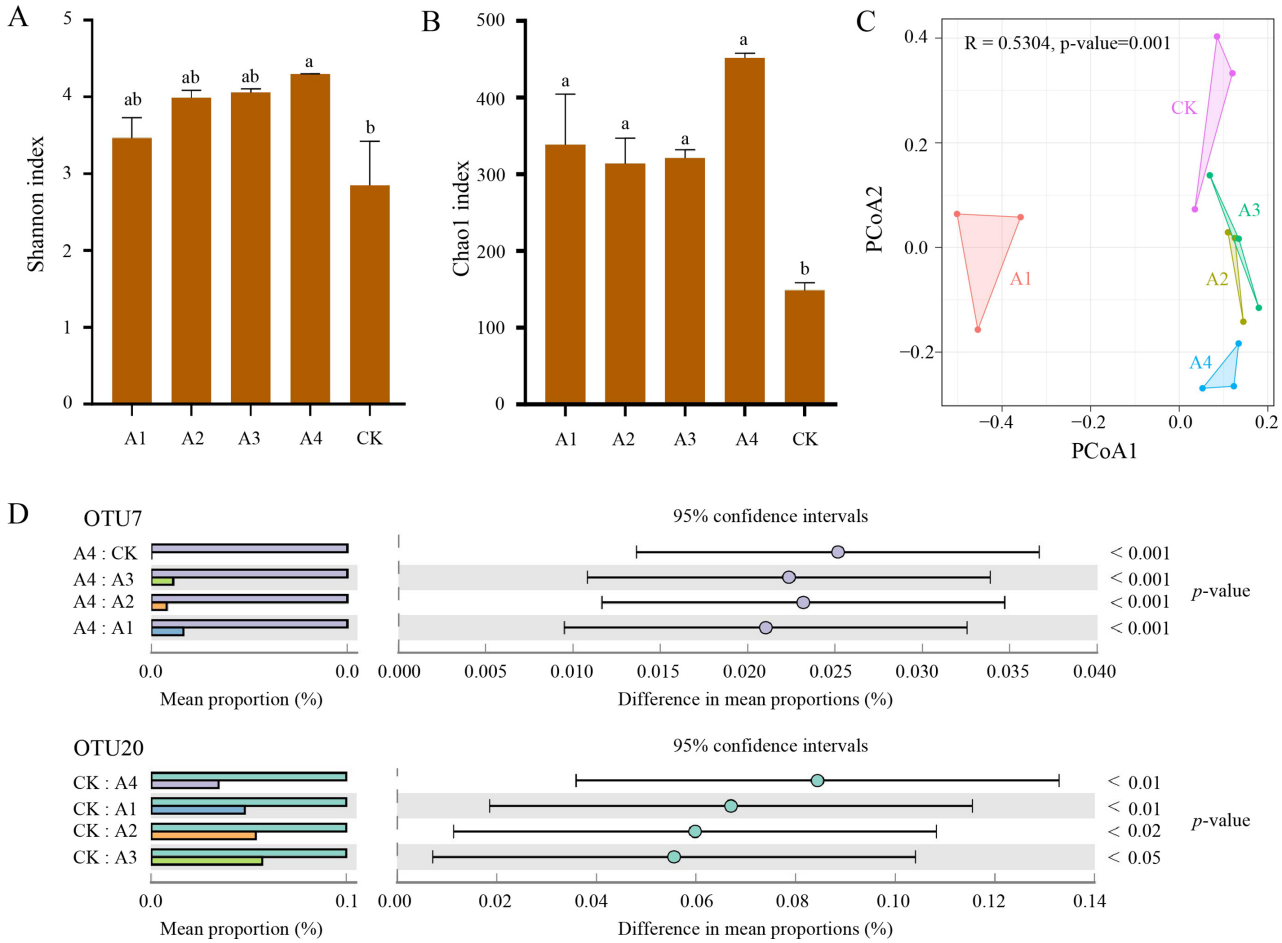
Diversity of soil microbial community structure under different mulching practices. **(A,B)** Alpha diversity analysis of five soil samples (**A**: Shannon index; (**B**: Chao1 index). One-way ANNOVA was used to analyze differences between groups (*p* < 0.05). **(C)** Beta diversity analysis of five soil samples (Principal Coordinate Analysis, PCoA, based on Bray-Curtis distances). **(D)** Bar plots showing intergroup differences in the relative abundance of *Candidatus Saccharibacteria* and *Thaumarchaeota*. The left panel displays bar plots comparing the mean abundance of species, while the right panel shows the mean abundance with 95% confidence intervals across groups.

To investigate the effects of different mulching treatments on soil microbial community diversity and structure, this study constructed microbial rarefaction curves based on OTU tables. The results showed that as sequencing depth increased, the curves of all samples plateaued, indicating that the sequencing depth was sufficient to meet the requirements for subsequent analyses (Supplementary Figure S2). Further analysis using Alpha diversity indices (Shannon index and Chao1 index) revealed that the bacterial community richness and diversity in the CK group were significantly lower than those in mulch treatment groups, while the A4 group exhibited the highest bacterial community richness and diversity (Figures 2A,B; Supplementary Table S3). Principal Coordinate Analysis (PCoA) based on Bray-Curtis distances demonstrated significant differences in soil bacterial community structure among different mulching treatments, with the A1 group showing distinct separation from the other four groups (Figure 2C). Additionally, the relative abundance of Candidatus Saccharibacteria was significantly higher in the A4 group compared to other treatments, while the relative abundance of Thaumarchaeota, a group of archaea, was significantly higher in the CK group (Figure 2D).

### 3.3 Bacterial communities sensitive to different mulching types

**Figure 3:**
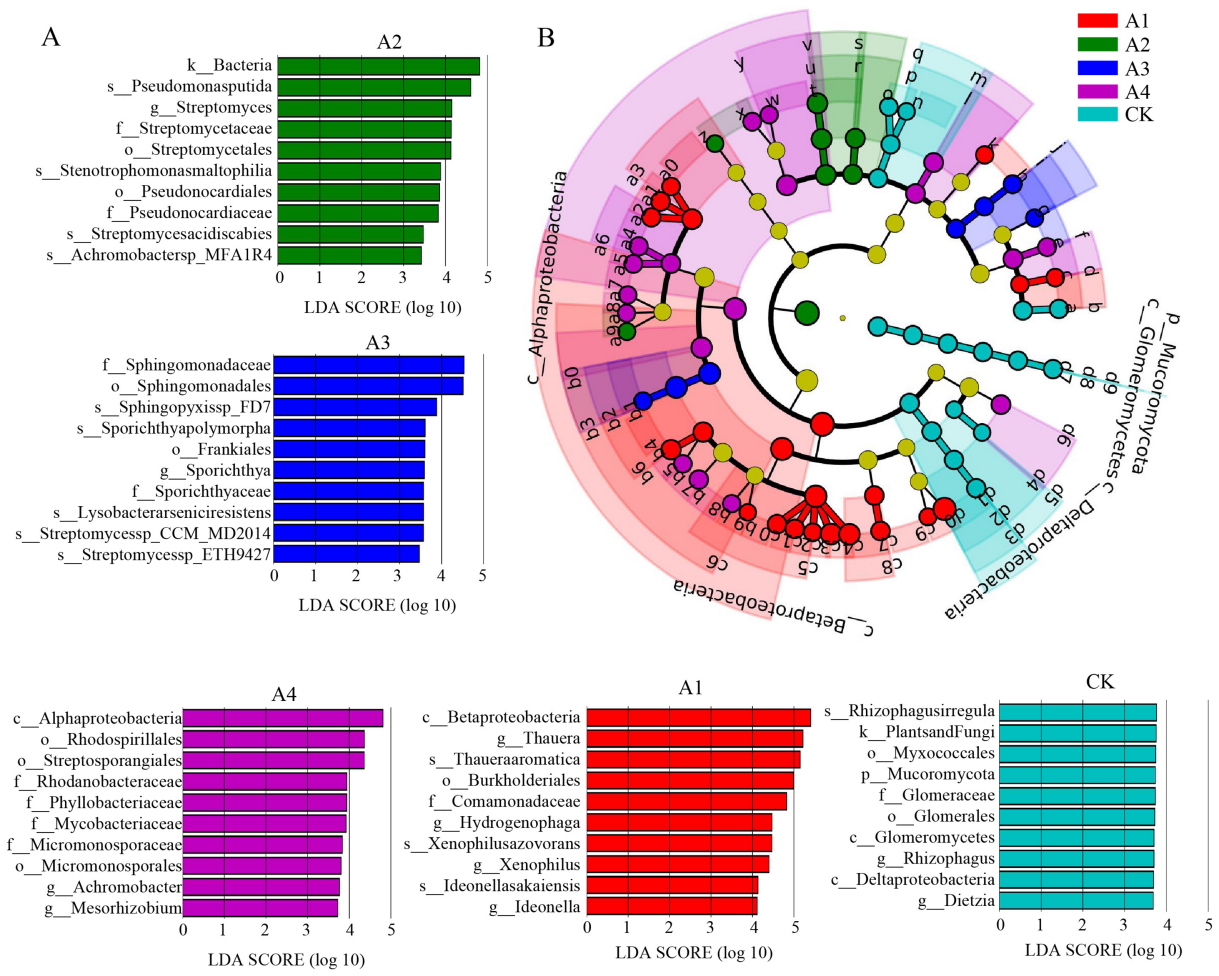
Intergroup differences among five soil samples (LEfSe). **(A)** Linear discriminant analysis (LDA) score plot showing the significantly different taxa, with the top 10 taxa ranked by LDA score for each treatment; **(B)** cladogram illustrating the phylogenetic distribution of microbial taxa with significant intergroup differences. The taxonomic annotations of the nodes in the phylogenetic tree are provided in Supplementary Table S5.

To identify significant differences in soil microbial community characteristics under different cover treatments, this study used LEfSe for biomarker analysis. The results showed that 66 bacterial taxa differed significantly across the five soil sample groups, with the A1 treatment group exhibiting the most differentially expressed taxa (21) and the A2 treatment group the fewest (7) (Figure 3A). Notably, compared to other groups, A1 and A4 showed some commonalities in their microbial community changes. For example, both groups contained Rhizobiales bacteria (A1: Bradyrhizobiaceae; A4: Phyllobacteriaceae) (Figure 3B; Supplementary Table S4). Besides these shared bacteria, five genera of the family Cladosporiae (*Acidobacteria*, *Cyclosporia*, *Deazobacterium*, *Hydrogenobacteria*, and *Heterotrophs*) in A1 showed significant differences, and these bacteria have been shown to play important regulatory roles in soil nitrogen metabolism. In conclusion, LEfSe analysis indicates that different cover treatments significantly affected the soil microbial community structure by altering key microbial taxa (such as Mucoromycota and Comamonadaceae).

### 3.4 Soil microbial community interaction networks

**Figure 4:**
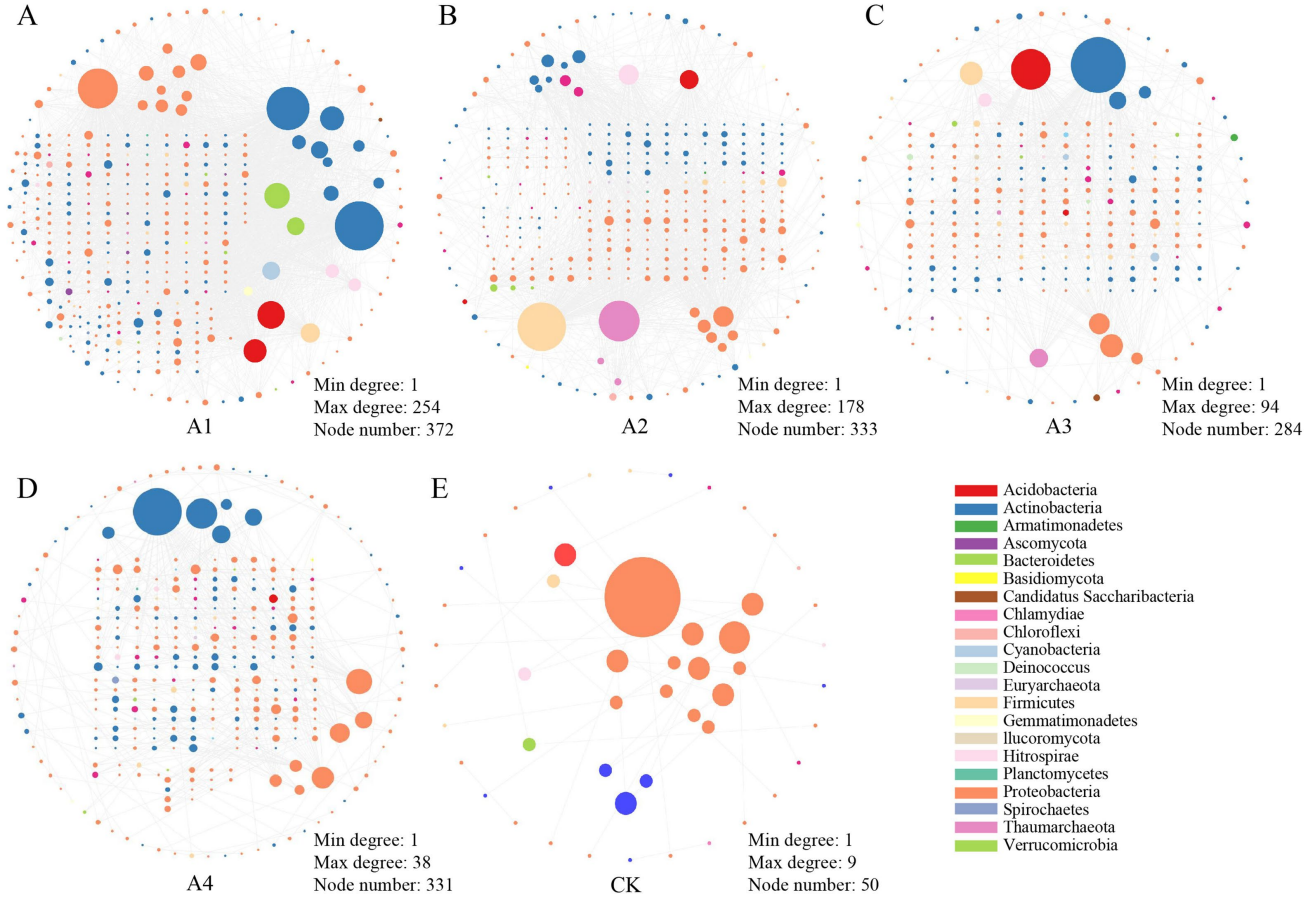
Overview of microbial community network topology for each treatment group. From left to right, the microbial interaction networks for the A1 **(A)**, A2 **(B)**, A3 **(C)**, A4 **(D)**, and CK **(E)** groups are shown. The size of each node is proportional to its degree (number of connections), and the color represents different microbial phyla.

To elucidate the effects of straw and plastic mulching treatments on soil microbial community structure, this study constructed microbial interaction networks to evaluate the symbiotic patterns of soil microbial populations under different mulching conditions (Figure 4). Network analysis revealed that Proteobacteria and Actinobacteria were the two dominant phyla constituting the network structure. The complexity of the networks was further assessed by analyzing topological features, including the number of nodes, edges, average degree, average path length, clustering coefficient, and modularity. The results showed that the network constructed for the CK group was the simplest, containing only 50 nodes and 43 edges. In contrast, the network for the A1 treatment group exhibited the highest complexity, with 372 nodes and 2,599 edges. This suggests that different soil cover treatments significantly altered the complexity of the microbial co-occurrence network. Specifically, the clustering coefficient of the actual network was significantly higher than that of the random network, indicating that the microbial communities formed a non-random co-occurrence pattern with a highly modular structure, revealing stable and close ecological interactions between species (Supplementary Table S6). Furthermore, centrality analysis identified key species with high connectivity in different treatment groups: *Mycolicibacterium obuense* in A1, *Paenibacillus massiliensis* in A2, *Microbacterium* sp. LKL04 in A3, and *Micromonospora chersina* in A4. Notably, no nodes with significantly high connectivity were observed in the CK group. These findings provide new insights into the effects of different mulching treatments on soil microbial interaction networks.

### 3.5 Functional prediction of soil microbial communities

**Figure 5:**
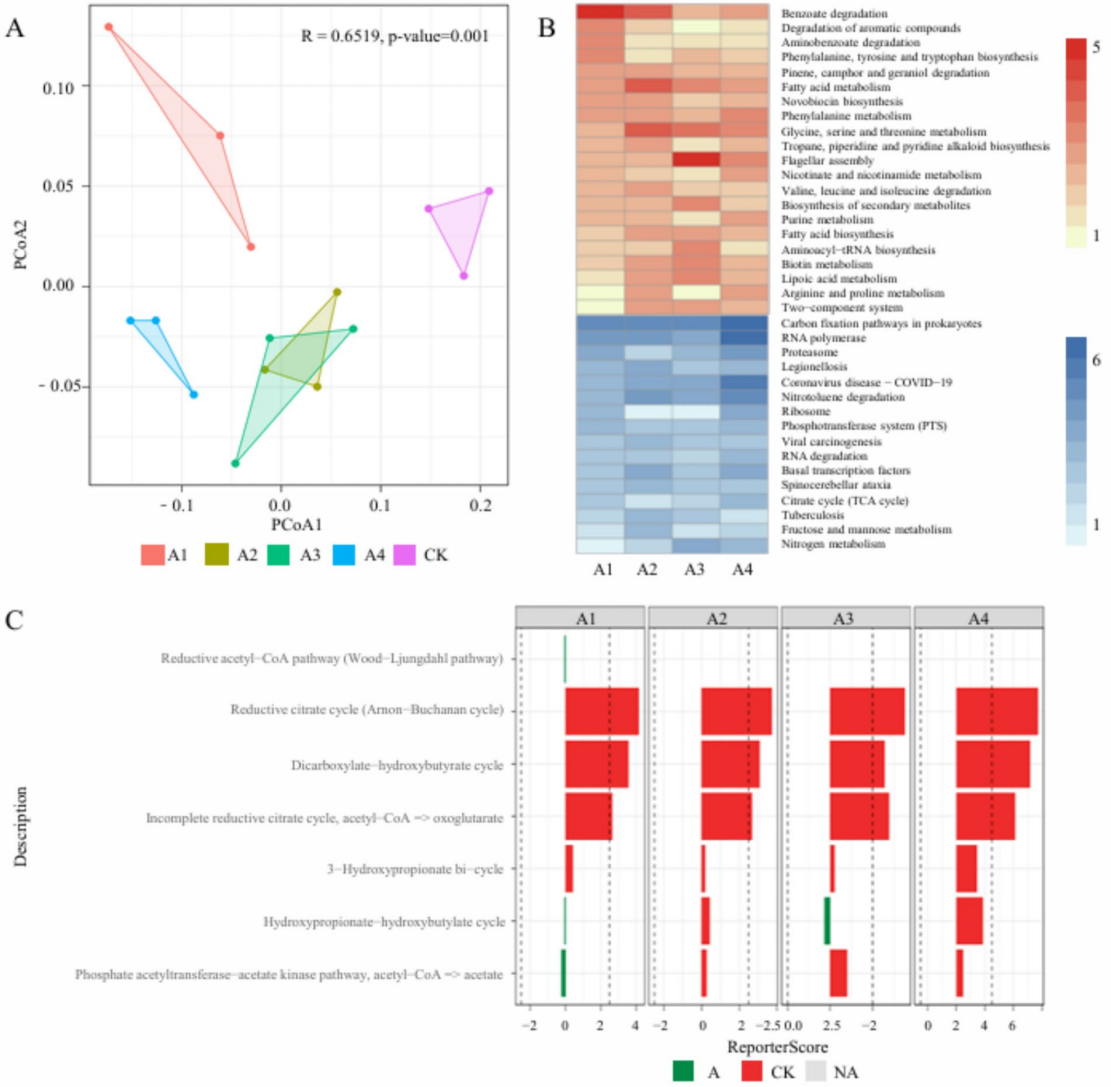
Functional annotation of soil microbial communities. **(A)** PCoA based on KO gene abundance, showing the clustering of functional composition in soil microbial communities under different treatment groups; **(B)** clustered heatmap based on ReportScore, where the *x*-axis represents the four treatment groups and the *y*-axis represents KEGG pathways. A ReportScore > 0 indicates that the pathway is enriched in the CK group, while a ReportScore < 0 indicates that the pathway is depleted in the CK group; **(C)** this demonstrates the enrichment or depletion results of each group in different modules of the carbon fixation pathway in prokaryotes compared to the control group.

To investigate the effects of different mulching treatments on soil microbial functional genes, this study analyzed the compositional differences of functional genes based on the KO database, identifying a total of 4,394 KO genes (Supplementary Table S7). PCoA based on Bray-Curtis distances was used to compare the similarity of functional genes among different treatment groups (Figure 5A). The results revealed significant differences in the functional composition of soil microbial communities under different mulching treatments, with the two non-degradable mulch treatment groups (A2 and A3) showing similar functional profiles. Further KO enrichment analysis indicated that, compared to the CK, all mulching treatment groups exhibited similar trends in functional pathway changes. Specifically, the abundances of genes related to benzoate degradation, degradation of aromatic compounds, and aminobenzoate degradation were significantly increased after mulching treatments. In contrast, the abundances of genes associated with carbon fixation pathways in prokaryotes, RNA polymerase, and proteasome showed a decreasing trend (Figure 5B; Supplementary Table S8). We further calculated the ReporterScore of relevant modules in the Carbon fixation pathways in prokaryotes. The results showed that the Reductive citrate cycle (Arnon-Buchanan cycle), Dicarboxylate-hydroxybutyrate cycle, and Incomplete Reductive citrate cycle (acetyl-CoA = > oxoglutarate) are the three main modules depleted in this pathway (Figure 5C). These results indicate that different mulching measures influence key metabolic processes such as organic matter degradation and carbon fixation in the soil by altering the functional gene composition of the microbial community.

### 3.6 Relationship between microbial communities and physicochemical properties

**Figure 6:**
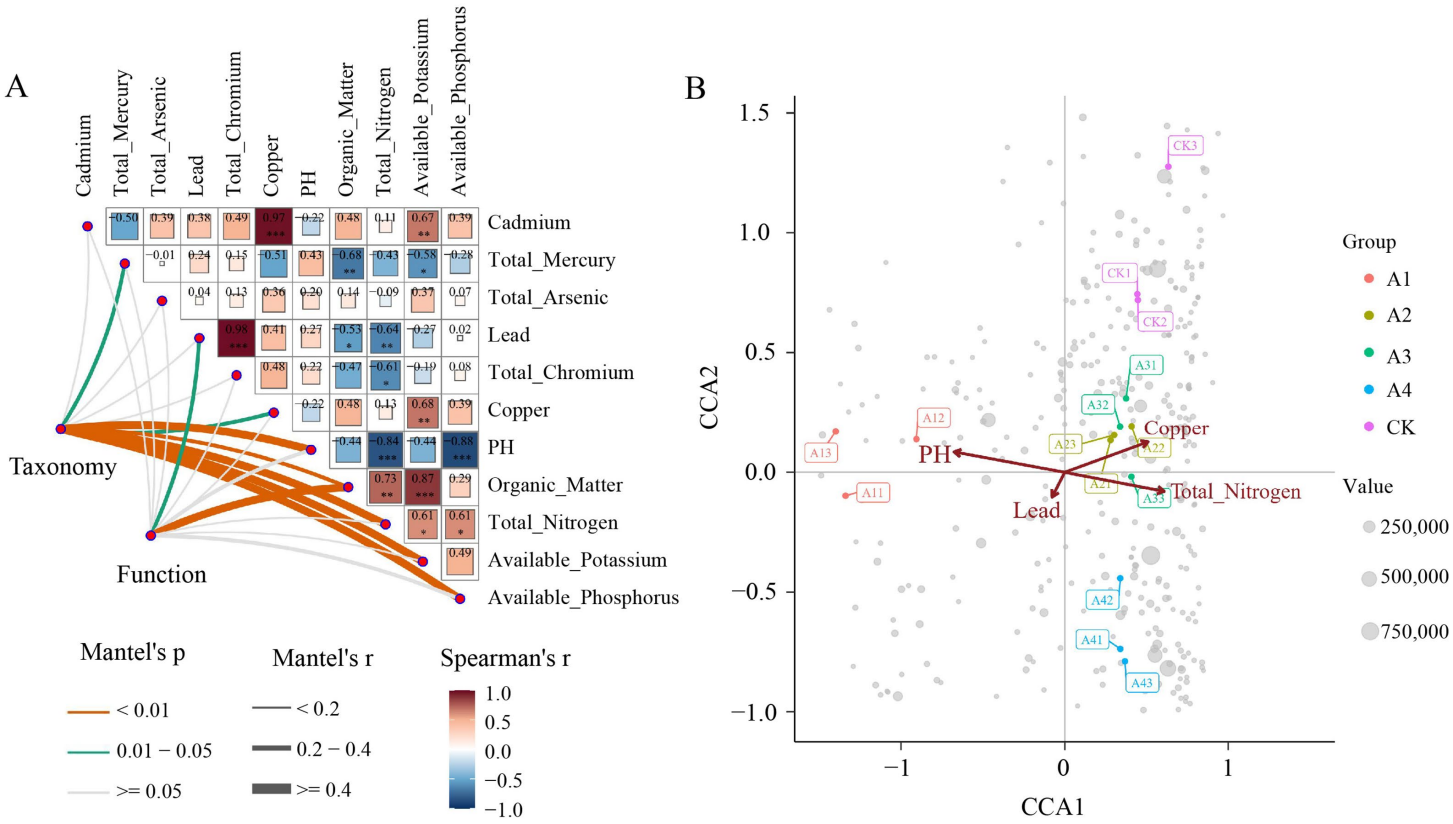
Relationship between soil microorganisms and environmental factors. **(A)** Mantel correlation analysis between microbial abundance (taxonomic level) and functional genes (functional level) with environmental factors; **(B)** CCA of microbial communities based on four environmental factors (pH, lead, copper, and total nitrogen).

To investigate the associations between environmental factors and soil microbial communities, this study conducted Mantel correlation analysis (Figure 6A; Supplementary Table S9). The results revealed significant correlations among environmental factors: pH showed a strong negative correlation with total nitrogen and available phosphorus, while copper exhibited a strong positive correlation with cadmium, and total chromium showed a strong positive correlation with lead. Additionally, four environmental factors (pH, total nitrogen, available potassium, and available phosphorus) were significantly correlated with microbial abundance (*r* > 0.4, *p* < 0.01), and organic matter was significantly correlated with microbial function (*r* > 0.4, *p* < 0.01) (Supplementary Table S10). To more accurately assess the impact of environmental factors on each treatment group, we used Vegan screening to identify four representative environmental factors (pH, lead, copper, and total nitrogen) and performed CCA. The results showed that, under the influence of these environmental factors, the 15 samples could be divided into three categories. Treatment groups A2 and A3 were mainly positively correlated with copper and total nitrogen, while treatment group A1 was mainly positively correlated with pH (Figure 6B).

## 4 Discussion

Mulching treatments can significantly alter the composition of soil microbial communities (Yang et al., 2022). In this study, compared to non-mulching treatments, soil microbial content increased to varying degrees after mulching, with the straw mulching and biodegradable mulch treatment groups showing significantly higher numbers of unique OTUs than CK. Notably, the abundance of the Enterobacter genus decreased to varying degrees after mulching. Enterobacter is not only widely present in rhizosphere soil but also within plant tissues, promoting the growth of various crops (e.g., wheat, peas, citrus, corn, bananas) and is recognized as a plant growth promoter (Khalifa et al., 2016; Macedo-Raygoza et al., 2019). For example, Liu et al. found that the Enterobacter strain N10 significantly increased the root length and plant height of sunflowers and wheat, making it one of the most effective rhizobacteria for wheat yield enhancement (Liu et al., 2016). Additionally, Li et al. demonstrated that *E. cloacae HSNJ4* promoted seed germination in rapeseed, increasing root length, stem length, lateral root number, chlorophyll content, and salt tolerance (Li et al., 2017). In summary, the mulching treatments not only significantly increased the total microbial abundance and specific groups (such as straw and biodegradable plastic mulch treatments), but also significantly reduced the abundance of Enterobacter species, which have growth-promoting functions, revealing the multidimensional impact of mulching measures on soil microbial communities. On the other hand, this study observed that the biodegradable mulching treatment (A1) significantly enhanced the relative abundance of *Thauera*. As a typical denitrifying bacterium, its metabolic activity is highly dependent on the availability of sufficient organic carbon sources and anaerobic conditions (Yuan et al., 2021; Wang et al., 2024). The PBAT/PLA-based cover materials used in this study continuously release organic carbon during the degradation process, while their physical coverage effect may also improve the anaerobic microenvironment in the soil, collectively creating favorable conditions for the proliferation of *Thauera*. This finding provides a new perspective for elucidating the underlying mechanisms by which biodegradable cover materials regulate soil microbial functional groups.

After mulching treatments, the species richness and diversity of soil microorganisms increased to varying degrees. Mulching influences soil microbial diversity and richness by altering soil moisture, temperature, and nutrient conditions (Gan et al., 2013; Yang et al., 2024). Additionally, different mulching practices significantly impact microbial community structure. Compared to plastic film mulching, organic mulching provides a nutrient-rich substrate, thereby enhancing soil microbial richness and altering community structure. Studies have shown that soil microbial diversity under bark mulching is significantly higher than under gravel mulching or in unmanaged fields (Martir-Torres and Bruns, 2013). In this study, the richness and diversity of soil microorganisms were similar under the two plastic film mulching treatments, with comparable community structures. In contrast, the microbial community composition under biodegradable mulch and straw mulching treatments differed significantly from that under plastic film mulching. This indicates that organic and inorganic mulching practices establish distinct soil microbial ecological networks during chieh-qua cultivation. Furthermore, the abundance of Candidatus Saccharibacteria was significantly higher in the A4 treatment group compared to other groups. Candidatus Saccharibacteria is a dominant bacterial group commonly found in grassland ecosystems, suggesting that straw mulching may be the primary reason for its increased abundance in the A4 group. However, the abundance of Thaumarchaeota was significantly higher in the CK group than in other treatment groups. Thaumarchaeota, a group of autotrophic ammonia-oxidizing archaea, plays a crucial role in aerobic nitrification and the global nitrogen cycle (Limpiyakorn et al., 2005; Brochier-Armanet et al., 2008). This phenomenon may be attributed to the reduced soil oxygen levels under mulching treatments, which likely inhibited their growth.

LEfSe analysis revealed notable differences in both fungal and bacterial biomarkers. The fungal genus Rhizophagus (phylum Mucoromycota) was significantly enriched in the CK group, while the A1 treatment exhibited a higher number of biomarkers from the bacterial family Comamonadaceae, including *Acidovorax, Alicycliphilus*, *Diaphorobacter, Hydrogenophaga*, and *Xenophilus*. Fungi such as *Rhizophagus* and plant growth-promoting rhizobacteria are important for regulating soil-borne diseases and nutrient availability; for example, inoculation with *Funneliformis mosseae* has been shown to reduce *Fusarium oxysporum* abundance and to affect plant growth, disease severity, and root/rhizosphere microbial composition under continuous cropping (Jie et al., 2015, 2019a,b). In addition, the Comamonadaceae genera identified in A1 are known to participate in denitrification and to influence nitrogen metabolism (Patel et al., 2021; Fei et al., 2024; Ma et al., 2024). Combined with the earlier finding of the *Thauera* genus, the results suggest that denitrifying bacteria dominate the A1 treatment group. The denitrification process converts inorganic nitrogen in the soil into N_2_, potentially leading to nitrogen fertilizer loss. This process requires organic carbon sources, and studies have shown that certain biodegradable polymers, such as polycaprolactone (PCL), polybutylene succinate (PBS), polylactic acid, and polyhydroxyalkanoates, serve as primary organic carbon sources in denitrification (Honda and Osawa, 2002; Shen and Wang, 2011; Zhang et al., 2016). It was reported that using wood chips, barley straw, and cardboard as carbon sources and media could remove 67–89% of nitrate from groundwater through solid-phase denitrification (Healy et al., 2012). Therefore, although biodegradable mulch can replace plastic mulch to reduce environmental pollution, the organic carbon released during its degradation may promote the proliferation of denitrifying bacteria, leading to the conversion of inorganic nitrogen into N_2_ and subsequent nitrogen fertilizer loss. Based on these findings, we recommend supplementing nitrogen fertilizer promptly after applying biodegradable mulch to maintain soil fertility.

Soil microorganisms exhibit complex interactions, and co-occurrence network analysis has revealed the association patterns of soil microbial communities under different mulching practices (Zhang et al., 2024). This study found that biodegradable mulch significantly enhanced the correlations among soil microorganisms. This study confirms that biodegradable cover treatments significantly enhance the connectivity between soil microorganisms, supporting the existing understanding that they can increase the complexity of microbial networks (Sun et al., 2015). The underlying mechanism lies in the fact that the mulching measures optimize soil nutrient content, stimulate root growth, and thereby create more favorable environmental conditions for microbial community colonization and synergistic interactions. Additionally, biodegradable mulch significantly increases the abundance of microplastics in the soil, a change that may enhance microbial interactions and provide higher carbon sources (e.g., soil organic carbon, SOC; dissolved organic carbon, DOC), thereby further improving microbial network connectivity (Ives and Carpenter, 2007; Wang et al., 2018; Kelly et al., 2022). Furthermore, compared to the CK, the mulching treatment groups showed a significant reduction in metabolic pathways related to energy metabolism and carbon metabolism. Specifically, the expression of genes associated with energy metabolism pathways (e.g., Arnon-Buchanan cycle, AasP cycle, and the incomplete reductive citrate cycle) was significantly lower in mulched soils. Numerous studies have shown that metabolic pathways such as the anon-buchanan cycle are closely related to microbial carbon fixation. For example, the non-Buchanan cycle has been shown to enable energy acquisition from sulfides and facilitate carbon fixation (Gabrielsen, 2017; Ivanovsky et al., 2022). The reduced expression of genes on these prokaryotic carbon fixation pathways suggests that the mulched soil environment contains more readily available organic carbon sources, thereby reducing microbial reliance on energy-intensive carbon fixation pathways. These findings provide new insights into the potential impacts of biodegradable mulch on soil microbial ecosystems and offer directions for future research.

Our study demonstrates that different types of mulching films have distinct effects on soil microbial community structure. Biodegradable mulch significantly increased bacterial community diversity and enhanced the complexity and connectivity of microbial networks. In soils treated with biodegradable mulch, the abundance of denitrifying bacteria was markedly higher, while pathways related to energy metabolism and carbon metabolism were significantly reduced. Unlike traditional plastic films, the degradation of biodegradable mulch releases substantial amounts of organic carbon, which may enhance soil denitrification capacity and lead to the loss of inorganic nitrogen from the soil. These findings provide new insights into the impacts of biodegradable mulch on soil microbiomes and nitrogen cycling.

## Data Availability

The data are presented in the manuscript and the supporting materials. The raw reads data are submitted to the Short Read Archive (SRA) and BioProject accession number PRJNA1242032.

## Footnotes

1
http://huttenhower.sph.harvard.edu/galaxy/

2
https://r.igraph.org/

## Supplementary material

SUPPLEMENTARY FIGURE S1 Krona chart showing the taxonomic distribution of all microbiota.

SUPPLEMENTARY FIGURE S2 Rarefaction curves of microbial abundance in five soil samples.
