# Correction: Effects of different mulching practices on soil microbial community structure, function, and interaction networks in a chieh-qua cultivation

**DOI:** 10.3389/fmicb.2026.1832275

**Published:** 2026-03-27

**Authors:** Yan-chun Qiao, Xiao-xin Jiang, Jian-po Zhan, Xiao-hui Cheng, Feng Liu, Wen-sheng Zhang, Guo-ping He, Jia-zhu Peng, Yu-jun Wu, Song-guang Yang

**Affiliations:** 1 Guangzhou Academy of Agricultural and Rural Sciences, Guangzhou, China; 2 College of Horticulture, South China Agricultural University, Guangzhou, China; 3 Guangdong Key Laboratory for New Technology Research of Vegetables, Vegetable Research Institute, Guangdong Academy of Agricultural Sciences, Guangzhou, China

**Keywords:** chieh-qua, metagenomic, microbial community, mulching, soil

Authors [Jian-po Zhan, Xiao-hui Cheng] were erroneously spelled as [Jian-po Zan, Xiao-hui Chen].

The original version of this article has been updated.

