# Supplementary figures and images for: Effects of different mulching practices on soil microbial community structure, function, and interaction networks in a chieh-qua cultivation
Source: Front Microbiol. 2026 Feb 4;17:1691984. doi: 10.3389/fmicb.2026.1691984 (PMC12913568; doi:10.3389/fmicb.2026.1691984)

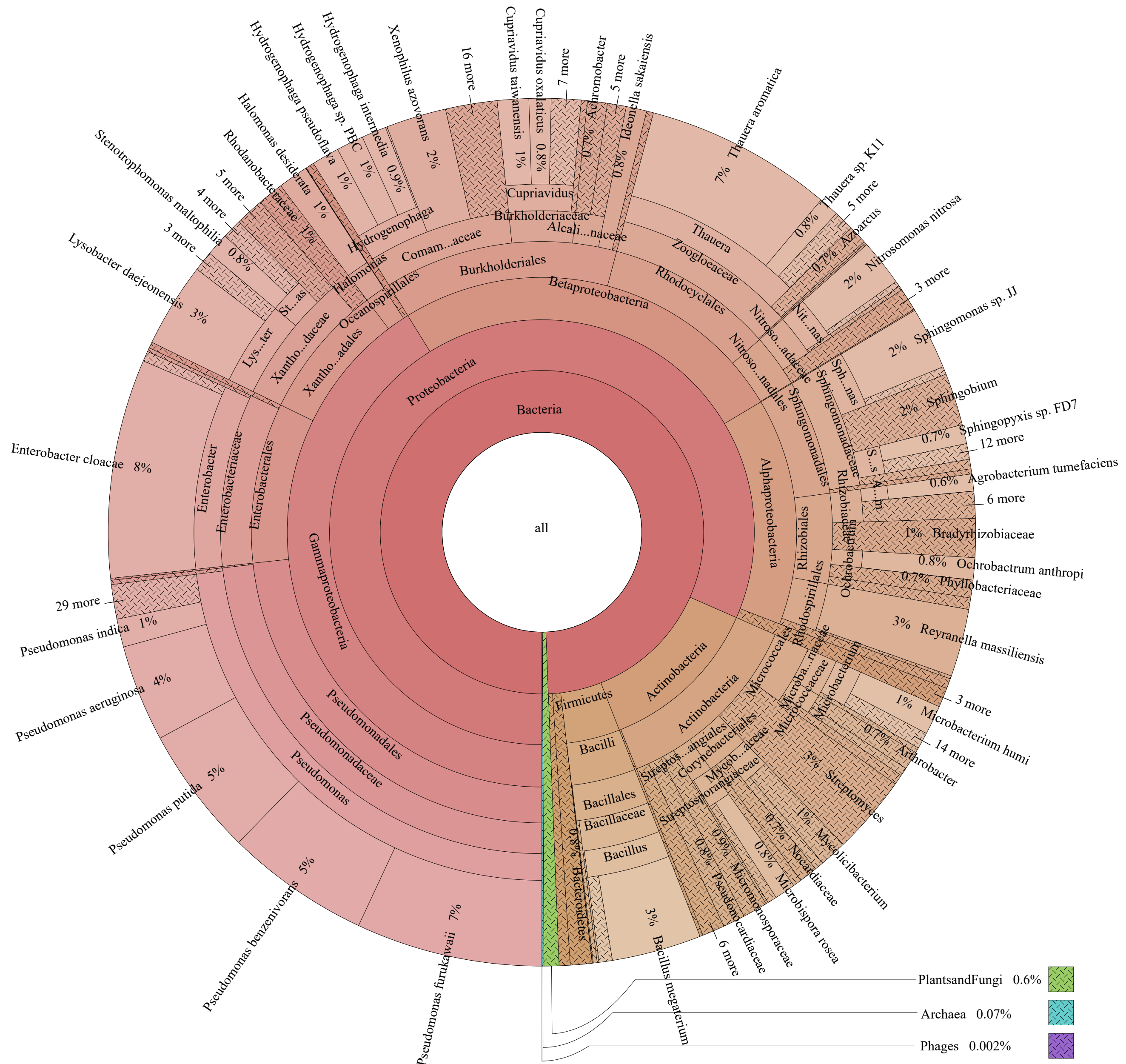

PlantsandFungi 0.6%

Archaea 0.07%

Phages 0.002%

Supplement: SUPPLEMENTARY FIGURE S1 — Krona chart showing the taxonomic distribution of all microbiota. [file Data_Sheet_1.pdf]

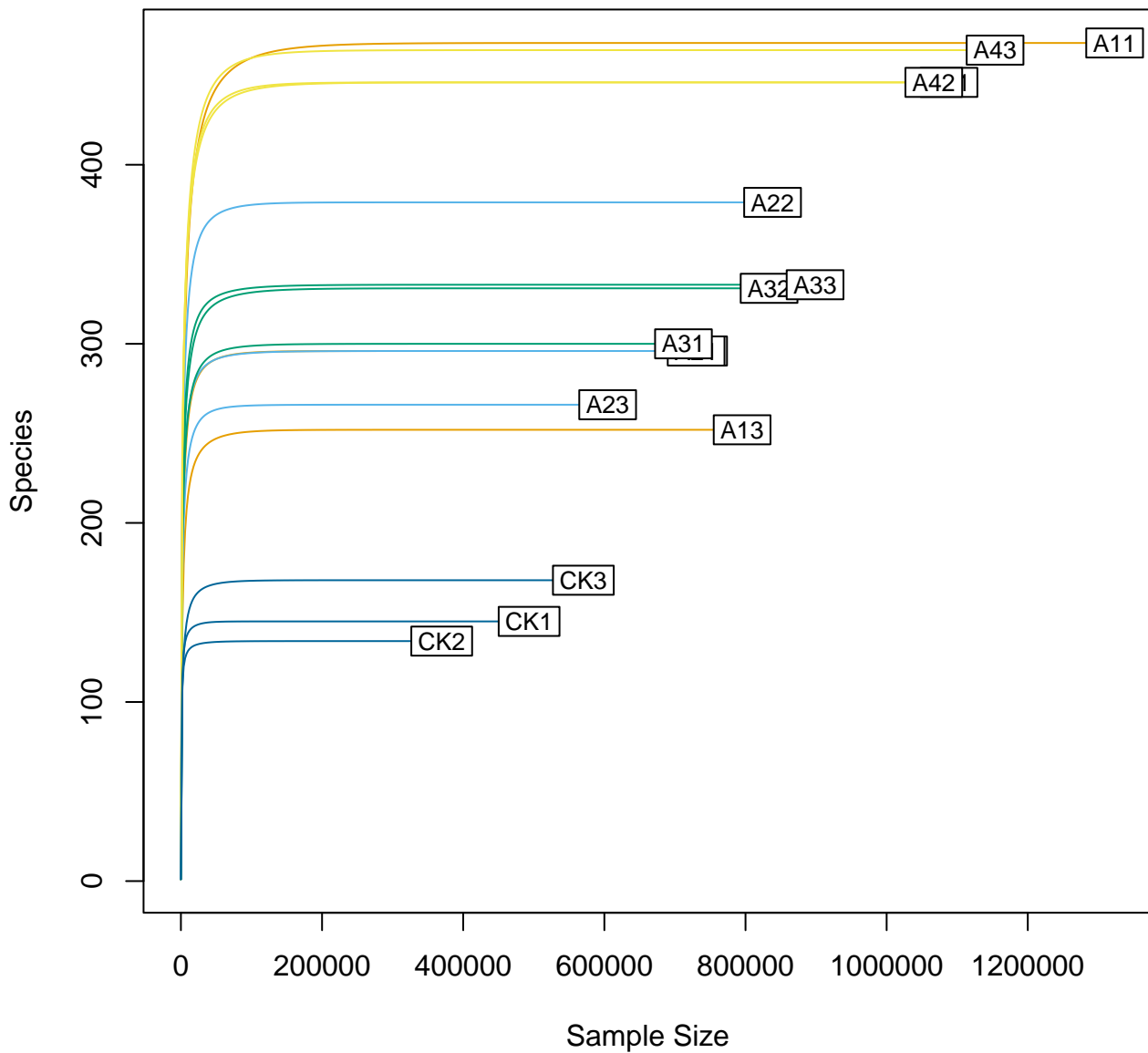

Supplement: SUPPLEMENTARY FIGURE S2 — Rarefaction curves of microbial abundance in five soil samples. [file Data_Sheet_2.pdf]
